# Comparison of different machine learning classification models for predicting deep vein thrombosis in lower extremity fractures

**DOI:** 10.1038/s41598-024-57711-w

**Published:** 2024-03-22

**Authors:** Conghui Wei, Jialiang Wang, Pengfei Yu, Ang Li, Ziying Xiong, Zhen Yuan, Lingling Yu, Jun Luo

**Affiliations:** Department of Rehabilitation Medicine, Second Affiliated Hospital of Nanchang University, Nanchang, 330006 Jiangxi People’s Republic of China

**Keywords:** Deep vein thrombosis, Lower extremity fractures, Machine learning, Prediction model, Diseases, Risk factors

## Abstract

Deep vein thrombosis (DVT) is a common complication in patients with lower extremity fractures. Once it occurs, it will seriously affect the quality of life and postoperative recovery of patients. Therefore, early prediction and prevention of DVT can effectively improve the prognosis of patients. This study constructed different machine learning models to explore their effectiveness in predicting DVT. Five prediction models were applied to the study, including Extreme Gradient Boosting (XGBoost) model, Logistic Regression (LR) model, RandomForest (RF) model, Multilayer Perceptron (MLP) model, and Support Vector Machine (SVM) model. Afterwards, the performance of the obtained prediction models was evaluated by area under the curve (AUC), accuracy, sensitivity, specificity, F1 score, and Kappa. The prediction performances of the models based on machine learning are as follows: XGBoost model (AUC = 0.979, accuracy = 0.931), LR model (AUC = 0.821, accuracy = 0.758), RF model (AUC = 0.970, accuracy = 0.921), MLP model (AUC = 0.830, accuracy = 0.756), SVM model (AUC = 0.713, accuracy = 0.661). On our data set, the XGBoost model has the best performance. However, the model still needs external verification research before clinical application.

## Introduction

DVT is a common perioperative complication in fracture patients and the incidence of postoperative DVT is significantly higher than that before the operation^1^. It is the third leading cause of cardiovascular death worldwide and a major complication limiting recovery in patients with lower extremity fractures^2^. Moreover, it can cause symptoms such as limb swelling, pain, and limitations in motor function^3^. As the disease progresses, DVT can even lead to the occurrence of pulmonary embolism, increasing the risk of death^4^.

More and more studies have been dedicated to finding the relevant factors for the occurrence and development of DVT in patients with lower extremity fractures during hospitalization, including age, underlying diseases, fracture types, operation time, bed rest time, coagulation function, etc.^5–7^. These studies promoted a better understanding of the factors associated with increased risk of DVT. However, whether to take measures to prevent DVT is usually up to doctors’ experience and patients’ symptoms. This approach, although clinically feasible, may delay the prevention of and treatment for DVT. Therefore, the identification of high-risk patients with DVT and timely prevention can avoid its occurrence.

Determining the important predictors of DVT is the key to accurately identify high-risk patients. However, the occurrence and development of DVT is influenced by many factors^8^. The occurrence of DVT is characterized by diversity and instability, and its influencing factors are still in the stage of continuous exploration. As a quantitative tool for disease risk assessment, the disease prediction model is an important auxiliary tool for identifying high-risk groups in the current clinical diagnosis, treatment and nursing process^9^. An in-depth analysis of the interaction mechanism of disease-related factors is the core of accurate disease prediction.

Machine learning is a scientific discipline that studies how computers learn from data. Through the computer algorithm, it automatically learns knowledge and rules from the data, so as to simulate human intelligent behavior and then achieve a certain purpose of technology and methods. Although there are currently scoring scales for predicting the risk of venous thrombosis in clinical practice, Mooney et al.^10^ demonstrated that machine learning is superior to traditional scoring methods in the accuracy of prediction. Shohat et al.^11^ also demonstrated that machine learning can predict venous thrombosis risk in hospitalized patients more accurately than scoring models developed in the past. Furthermore, Danilatou et al.^12^ considered machine learning as a method suitable for venous thromboembolism (VTE) risk prediction and believed that it has great advantages in finding the best predictors of VTE. At present, machine learning, as a new risk assessment and prediction tool, has been used by many researchers in clinical research and practice.

Ferroni et al.^13^ selected 1179 outpatient cancer patients who received chemotherapy but did not receive thrombus prophylaxis, and used the SVM model and randomized optimized multi-core learning model to predict DVT risk factors in these patients. The area under the Precision-Recall curve (AUCPR) of the model was 0.212. This study confirmed the possibility and effectiveness of machine learning method for DVT risk prediction. Ferroni et al.^14^ compared machine learning with Khorana score (KS) risk assessment scale, and the results showed that the predictive model was better than KS. The study conducted by James et al.^15^ aimed to measure the accuracy of the machine learning classifier for predicting DVT in cancer inpatients, and found that the RF model could correctly classify DVT and non-DVT patients with an accuracy of 74%. The study suggests that machine learning classifiers could be incorporated into electronic medical record systems that could provide an effective blood clot risk assessment for patients.

Although there are several prediction models for DVT, these prediction models have limitations^16,17^. Firstly, the inclusion and exclusion criteria of patients in the model were strict, and the population representative needed to be improved. Secondly, most machine learning algorithms were compared with traditional analysis methods, and the performance of multiple machine learning classifiers was not compared. Therefore, in this study, we compared the DVT prediction performance with several machine learning algorithms, such as XGB, LR, RF, MLP, and SVM models. Finally, we obtained the optimal model based on the comprehensive score.

## Methods

### Study population

The retrospective study was conducted according to STROBE guidelines and all methods were performed in accordance with the relevant guidelines and regulations. STROBE guidelines for an international, collaborative initiative of epidemiologists, methodologists, statisticians, researchers, and journal editors involved in the conduct and dissemination of observational studies, with the common aim of strengthening the reporting of observational studies in epidemiology. The Ethics Committee of the Second Affiliated Hospital of Nanchang University approved the study, without patient informed consent due to the anonymous nature of the data. Two specialists used the hospital digital medical record system to retrieve information about patients after lower limb fracture surgery in the Second Affiliated Hospital of Nanchang University from July 1, 2017 to July 1, 2023. Eligible patients were screened by checking medical records, doctor orders, and nursing records in the hospital’s digital medical record system. The results of patients with deep venous thrombosis of lower limbs were obtained through imaging examination reports in the hospital’s digital medical record system. All the obtained data were recorded into Excel software for sorting.

In this study, patients with lower extremity fractures who met the following criteria were included as eligible subjects: patients aged 18 years and older, with less than 20% missing items. Exclusion criteria are pathological fractures, fractures in other parts (such as sternum, and vertebrae), history of venous thromboembolism, oral contraceptives (nearly 1 month), pregnancy, and blood system diseases.

### Data preprocessing

With the occurrence or absence of DVT as the outcome index, all patients underwent color Doppler ultrasonography of both lower limbs. The sensitivity and specificity of color Doppler ultrasonography for DVT is more than 90%, which can determine the location and type of thrombus, determine the degree of embolization and collateral circulation, as well as evaluate the therapeutic effect^18^. DVT ultrasound diagnosis includes the following signs: enlarged lumen below the thrombus obstruction site, thickened vessel wall, solid echo in the lumen, filling defect of blood flow signal in the lumen, loss of phasic changes in blood flow spectrum, extrusion of the distal end increased blood flow to extremities disappears or weakens.

The multiple imputation (MI) was used for subjects with missing values. The basic idea of the MI is to infer multiple estimated fill values for missing values and generate multiple complete data sets for comprehensive analysis to determine the final estimated fill value^19^. The method modelled the actual posterior distribution of missing values through multiple estimates^20^. For outliers, they are reconfirmed by the data source and treated as null if they are still outliers after reconfirmation. The StandardScaler is used for standardization processing. The mean value of the data features processed by StandardScaler is 0, while the standard deviation is 1.

Data in the no-thrombosis cases outnumbered those in the thrombosis cases by a ratio of approximately 20:1. The Synthetic Minority Oversampling Technique (SMOTE) was applied to balance no-thrombosis and thrombosis groups. The SMOTE created new minority data by interpolation within the available minority data via bootstrap sampling and data generation via the k-nearest neighbors algorithm^21^. The K parameter, which determines the number of closest neighbors considered with each SMOTE iteration, was set to 5. To achieve an approximate balance between the no-thrombosis and thrombosis groups, 4010 new data for the thrombosis group were created.

### Selection of predictors

Based on a search of relevant literature and clinical judgment, we compiled a list of potential predictors of DVT. There are 36 predictors including sex, rh blood type, ABO blood type, smoking, alcohol consumption, operation time, hypoalbuminemia, kidney disease, cerebrovascular disease, atrial fibrillation, heart disease, cancer, chronic obstructive pulmonary disease (COPD), osteoporosis, hypertension, diabetes, lung infection, fracture type, surgical grade, age, total cholesterol (mmol/L), T triacylglycerol (mmol/L), free fatty acid (mmol/L), albumin (g/L), globulin ratio, calcium (mmol/L), potassium (mmol/L), platelets (10^12^/L), red blood cells (10^12^/L), white blood cells (10^9^/L), fibrinogen (g/L), D-dimer (mg/lFEU), international normalized ratio(INR), plasma prothrombin time (s), hospital stay (days) and C-reactive protein (mg/L). The fracture site was divided into hip, femur, tibia, and multiple fractures. Besides, the operation grade was divided into first-level operation, second-level operation, third-level operation, and fourth-level operation. The operation time was categorized into two subsets: those within three hours and the others above.

### Ranking of important predictors

Before building the models, we used the XGBoost model to calculate the average contribution value of each predictor, and listed the top 10 important predictors. Based on the study^22^, and in order to improve efficiency, we skipped the hyperparameter tuning that requires a lot of computational resources, and chose a set of relatively reasonable hyperparameter values in a more balanced way. The model parameters are: learning_rate = 0.1, maximum tree depth = 8, minimum fork weight sum = 4, L2 regularization coefficient = 0.5. The 10 most important variables in the XGBoost model (from high to low) are age, hypertension, fibrinogen, surgical grade, platelets, T triacylglycerol, free fatty acid, D-dimer, globulin ratio, and diabetes. See Table 1 for details.

**Table 1:** Ranking of important features of the XGBoost model.

Predictors	Total gain	Total cover	Gain	Cover	Weight importance
Age	3071.156	21,778.921	42.655	302.485	72
Hypertension	1660.922	6932.982	166.092	693.298	10
Fibrinogen	1033.768	11,259.496	17.229	187.658	60
Surgical grade	1004.936	7708.09	71.781	550.578	14
Platelets	864.13	5975.52	12.345	85.365	70
T triacylglycerol	757.919	7882.993	15.468	160.877	49
Free fatty acid	751.291	6974.667	12.118	112.495	62
d-dimer	688.785	6367.794	15.306	141.507	45
Globulin ratio	675.949	8519.171	14.695	185.199	46
Diabetes	671.389	6671.051	67.139	667.105	10

### Model building

The working principle of the XGBoost model is to re-adjust the training samples of the decision tree obtained from the initial training set, especially after further adjustment of the training samples that the decision tree got wrong, and then use them to train the next decision^23^. The max_depth was 6 and the learning_rate was 0.01 in the XGBoost model. The LR model is a generalized linear regression analysis model that can be extended to assess the correlation between various types of observed data and certain predictors^24^. The LR model used L2 loss for regularization, the liblinear solver as the optimizer, and the one-vs.-rest scheme as the loss function. The model was trained for 100 iterations and had a C-index of 1. The RF model is an algorithm that combines multiple decision trees through ensemble learning^25^. The RF model was trained with 20 decision trees with maximum tree depth of 10. The quality of split was measured using Gini impurity. The construction principle of the MLP model is to imitate the human brain, which consists of an input layer, an output layer, and hidden layers of 20 and 10^26^. The MLP model was trained with 20 iterations and ReLU activation. The SVM model maps the original data to a high-latitude space through a nonlinear function, in which the original data can be separated by a line called a separating hyperplane, and the distance from the data point to this line represents the prediction result of the model confidence (the farther the distance, the more confident the model is about the accuracy of the prediction)^27^. The hyperparameters of five models are as Table 2.

**Table 2:** Hyperparameters of different models.

Model	Hyperparameters
XGBoost	Objective: binary:logistic, learning_rate: 0.01, max_depth: 6, min_child_weight: 2, reg_lambda: 1
LR	C-index: 1, max_iter: 100, penalty: l2, tol: 0.0001
RF	Criterion:gini, max_depth:10, min_impurity_decrease:0.0, n_estimators:20
MLP	activation: ReLU, hidden_layer_sizes: (20, 10), max_iter: 20
SVM	C-index: 1.0, kernel: rbf, tol: 0.001

The 10 most important predictors used to train the models were encoded into the machine learning models. We then divided all the data into five equal parts and chose one of them as the test set. The remaining four parts were used as the training set and repeated five times. Five machine learning models, including XGBoost model, LR model, RF model, MLP model, and SVM model, were used for model construction by using fivefold cross-validation. Model building was done in R version 3.6.3 and python version 3.7. This work was supported by the Extreme Smart Analysis platform (https://www.xsmartanalysis.com/).

### Model evaluation

The confusion matrix is a cross-tabulation of real values through which multiple evaluation indexes of the model can be constructed. True Positive (TP) means that the true classification of the sample is positive, and the identification result of the model is also positive; False Negative (FN) means that the true classification of the sample is positive, but the model serves as a negative class. False Positives (FP) means that the true classification of the sample is a negative class, but the model will regard it as a positive class. True Negative (TN) means that the true classification of the sample is negative, and the identification result of the model is also negative.

The size of the area under the receiver operating characteristic (ROC) curve is AUC. AUC reflects the overall predictive performance of the model. The closer the AUC is to 1, the better the predictive performance of the model will be; Accuracy is used to describe the accuracy of the model, that is, the accurate number/total number of samples that the model can identify. The higher the accuracy of the model is, the better the effectiveness of the model will be. Sensitivity represents the ratio of the number of positive patients correctly identified in the model to the total number of positive patients. Specificity refers to the proportion of the number of correctly predicted negative patients in the number of true negative cases. The F1 score is the harmonic mean of accuracy and sensitivity and its value ranges from 0 to 1. The higher the F1 value is, the better the efficiency will be. The Kappa is used for consistency testing and measuring the effectiveness of classification. Consistency refers to whether the predicted results are consistent with the actual results. The calculation of the Kappa is based on the confusion matrix, with values ranging from − 1 to 1, usually greater than 0. When we obtained the AUC, accuracy, sensitivity, specificity, F1 score, and Kappa of five machine learning algorithms on the test set, the optimal model was selected by comprehensive score.

### Statistical analysis

Using DVT as the outcome indicator, the data were divided into two groups. The continuous variables such as age, were expressed as median and interquartile range (IQR). Gender and other variables were expressed as percentages (%). For classified variables, if the sample size > 40 and the expected frequency > 5, the Chi-square test would be used. For continuous variables, if the distribution is normal and the variance is homogeneous, the t-test would be used. The Welch’s t-test would be used when the normal distribution was met but the variance was not homogeneous. For non-normal distributions, the Mann–Whitney U test would be used. All Statistical analyses were performed using R version 3.6.3 and python version 3.7. *P* < 0.05 was considered to be statistically significant. This work was supported by the Extreme Smart Analysis platform (https://www.xsmartanalysis.com/).

### Ethical approval

The present research was approved by the Biomedical Research Ethics Committee of the Second Affiliated Hospital of Nanchang University (BR/AFISG-04/1.0). The informed consent was waived by the approving ethics committee due to the retrospective nature of the study.

## Results

### Patient demographic features

A total of 4424 patients were included in this study, including 4217 patients without DVT and 207 patients with DVT, the incidence rate of DVT was 4.68%. The average age was 61 years old, mainly between 45 and 76 years old. There were 2353 male patients included, accounting for 53.187% of the total, and 2071 females accounting for 46.813%. The incidence of DVT in patients with different parts of lower limb fractures was different (*P* < 0.001), and the incidence of DVT in patients with femoral fractures and multiple fractures was significantly higher than that of tibial and pelvic fractures. Since the number of first-level surgery patients in the no-thrombosis group was 0, the statistical result of the final surgery level was nan. Between the thrombosis group and the no-thrombosis group, the gender *(P* < 0.001), age (*P* < 0.001), ABO blood type (*P* < 0.001), hypoalbuminemia (*P* < 0.001), atrial fibrillation (*P* < 0.001), Total cholesterol (*P* < 0.001), T triacylglycerol (*P* = 0.002), albumin (*P* < 0.001), white blood cell ratio (*P* < 0.001), calcium ion (*P* < 0.001), platelets (*P* = 0.003), red blood cells (*P* < 0.001), fibrinogen (*P* < 0.001), d-dimer (*P* = 0.027), International Normalized Ratio (*P* = 0.036), C-reactive protein (*P* = 0.007) were statistically significant. There were no statistically significant differences in the remaining variables (see Supplementary Information).

### Model performance

In the test set, the AUC, accuracy, sensitivity, specificity, F1 score, and Kappa of the XGBoost model are 0.979, 0.931, 0.956, 0.911, 0.942, and 0.862. The AUC, accuracy, sensitivity, specificity, F1 score, and Kappa of the LR model are 0.821, 0.758, 0.803, 0.722, 0.764, and 0.516, respectively. The AUC, accuracy, sensitivity, specificity, F1 score, and Kappa of the RF model are 0.970, 0.921, 0.922, 0.921, 0.921, and 0.842. The AUC, accuracy, sensitivity, specificity, F1 score, and Kappa of the MLP model are 0.830, 0.756, 0.828, 0.704, 0.783, and 0.512, respectively. The AUC, accuracy, sensitivity, specificity, F1 score, and Kappa of the SVM model are 0.713, 0.661, 0.691, 0.642, 0.668, and 0.322. By comparing multiple indicators such as AUC, accuracy, sensitivity, specificity, F1 score, and Kappa, the XGBoost model performs best (see Table 3).

**Table 3:** The evaluation indicators of 5 models test set.

Model	AUC	Accuracy	Sensitivity	Specificity	F1 score	Kappa
XGBoost	0.979	0.931	0.956	0.911	0.942	0.862
LR	0.821	0.758	0.803	0.722	0.764	0.516
RF	0.970	0.921	0.922	0.921	0.921	0.842
MLP	0.830	0.756	0.828	0.704	0.783	0.512
SVM	0.713	0.661	0.691	0.642	0.668	0.322

**Figure 1:**
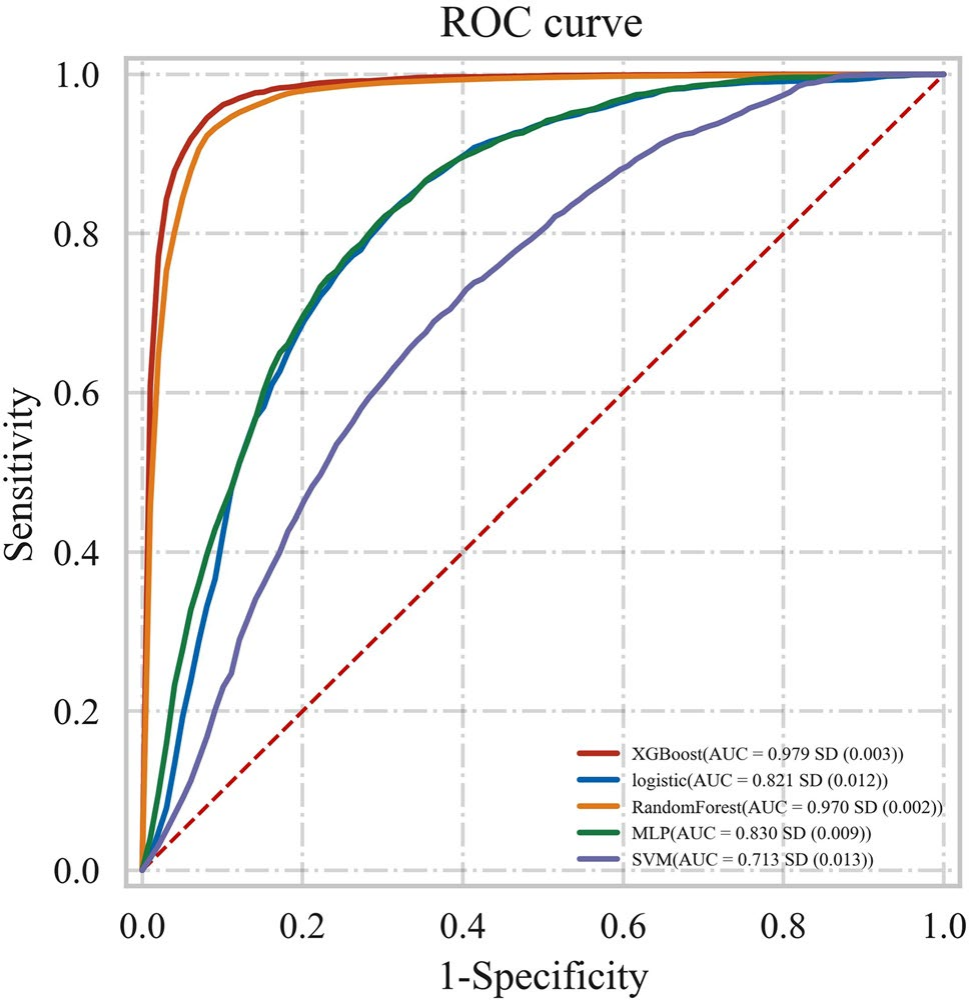
The ROC curve of test set for 5 models.

Figure 1 shows the ROC curves of the test set for these five models. The AUC values of the ROC curves of different models are the XGBoost model (0.979), LR model (0.821), RF model (0.970), MLP model (0.830), and SVM model (0.713). The XGBoost model and RF model are better than the other models on the test set.

## Discussion

By comparing the characteristic factors between the two groups for different thrombotic outcomes in patients with lower extremity fractures, we found sex, age, ABO blood type, hypoalbuminemia, atrial fibrillation, total cholesterol, T triacylglycerol, albumin, white blood cell ratio, calcium ion, platelets, red blood cells, fibrinogen, d dimer, international normalized ratio, C-reactive protein and fracture types were significantly different between the two groups (*P* < 0.05). The importance ranking (from high to low) of 10 predictors are age, hypertension, fibrinogen, surgical grade, platelets, T triacylglycerol, free fatty acid, d-dimer, globulin ratio, and diabetes. By comparing multiple indicators such as AUC, accuracy, sensitivity, specificity, F1 score, and Kappa, the XGBoost model has the best performance.

DVT is a common complication in patients with lower extremity fractures and may lead to fatal pulmonary embolism. The incidence of DVT in this study was 4.68%. Surgery is an independent risk factor for DVT in previous studies^28^. This is mainly because, during the operation, the long-term inability of the body to actively move and the trauma caused by the operation will lead to slow blood flow and damage to the blood vessel wall resulting in the formation of venous thrombosis. In addition, advanced age is also an independent risk factor for DVT^29^. For the elderly, not only the aging of vascular endothelial cells is accelerated, but also the vascular tension, permeability, and the regulation ability of the vascular wall are reduced, making vascular homeostasis difficult to maintain and more prone to thrombosis^30,31^.

Blood hypercoagulability is one of the mechanisms of thrombus formation, so coagulation function tests are closely related to thrombus formation. Previous studies have found a correlation between fibrinogen, INR, d-dimer, and thrombus^32,33^, which is consistent with our research results. High blood lipids and cholesterol will keep the body in a low-inflammatory state for a long time and increase blood viscosity^34^. Serum albumin, which has the function of inhibiting platelet aggregation and anticoagulation, has been found to be closely related to nutritional status and inflammatory response^35,36^. Studies found that serum albumin level was a predictor of left atrial thrombus in elderly patients with nonvalvular atrial fibrillation^37,38^. However, no research has found its correlation with DVT so far, and the relationship between them needs further verification by higher-quality research.

Blood type is an important characteristic of DVT in patients with lower extremity fractures. Li et al.^39^ found that patients with blood type B had a higher risk of DVT. Differently, Haddad et al. claimed that O blood type does not increase the chance of DVT. However, a higher-quality systematic review suggests that ABO blood type is closely related to the occurrence of DVT in cancer patients. The reason may be that ABO blood type is closely related to vWF level, which acts as a protective carrier of coagulation factor VIII and can promote the formation of thrombus^40^.

At present, more and more evidence has shown that there is a close relationship between inflammatory immune response and DVT^41,42^. In addition, the C-reactive protein, platelets, and neutrophils play an important role in the development of DVT^43^. However, it has been expounded in many relevant researches that severe trauma, stress sepsis and blood loss can all lead to systemic inflammatory response syndrome, which increases the reactivity of neutrophils and platelets in the body^44–46^. Neutrophils provide the original stimulation of DVT and recruit other cells in the coagulation process. Platelets produce circulating microparticles that accelerate blood coagulation^47^. Over the past few years, many scholars have investigated the association between inflammation and venous thrombosis. Kyril et al.^48^ analyzed all thrombosis patients in their Thrombosis Research Center and found that a high platelet-to-lymphocyte ratio would increase the risk of inducing DVT by 3 times.

In the past, most machine learning algorithms were compared with traditional analysis methods, and the performance of multiple machine learning classifiers was not compared. Therefore, we compare and analyze the prediction effects of five machine learning classifiers, and search for the optimal model by integrating AUC, accuracy, specificity, and other indicators. James et al.^15^ used the RF model to predict patients with venous thrombosis, and the accuracy rate was 74%, which was lower than that of the XGBoost model in this study. Liu et al.^49^ also used RF as a classifier to identify patients at high risk of thrombosis, and the AUC obtained by this method was between 0.77 and 0.79, which was also lower than the AUC obtained by the XGBoost model in this study. However, it is noteworthy that predictive factors varied in researches above, thus extra attention is required in this respect when comparing those models. Besides, the data is unavailable from the previous machine learning studies, so we cannot directly evaluate other machine learning methods. Several pivotal problems still require to be emphasized again including irregular data management, inappropriate data sources, and deficiency in algorithm details, which largely depends on the researchers' awareness and level of scientific research.

Overall, we analyzed various possible predictors of DVT and compared the predictive performance of various machine-learning methods. AUC is a performance measure of the ROC curve: the higher the AUC is, the higher the predictive power of the model is. By comparing multiple indicators such as AUC, accuracy, sensitivity, specificity, F1 score, and Kappa, the XGBoost model has the best performance. The XGBoost model is an optimized distributed gradient boosting library designed to be efficient, flexible, and portable. The XGBoost model fully considers the regularization problem by introducing the number of subtrees and the value of subtree leaf nodes in the loss function. In terms of efficiency, the XGBoost model has greatly improved the modeling efficiency by using the unique approximate regression tree bifurcation point estimation and sub-node parallelization, coupled with the characteristics of second-order convergence. So, its application is becoming more and more widespread, including prognosis and diagnosis of diseases, prediction of disease risk, etc. For example, Trakadis et al.^50^ used XGBoost analysis to identify people at risk of schizophrenia and found it to be the best performer in comparison with other algorithms, obtaining a better model efficacy (accuracy = 85.7, specificity = 86.6, sensitivity = 84.9, and AUC = 0.95). It can be seen that XGBoost as an efficient algorithm in machine learning is able to construct risk warning models well and achieve higher prediction accuracy.

The occurrence and development of DVT in the actual clinical environment is the result of the long-term interaction of multiple risk factors. The progress of disease is complex as well. The impact of predictors on the occurrence of DVT is judged solely by whether there is a statistical difference, which is different from the clinical situation and the pathophysiology of DVT. The machine learning algorithm can present the degree of influence of all predictors^51,52^. Therefore, the prediction model based on machine learning can fully reflect the impact of all predictors on DVT, and its results are closer to the actual clinical diagnosis and treatment.

We included variables possibly associated with DVT in the current algorithm to obtain accurate predictions. However, some limitations need to be considered when interpreting the findings of this study. First, there is a lack of detailed description of the variables regarding medication and lifestyle during hospitalization, such as antiplatelet, anticoagulant, antihypertensive, hypoglycemic, chemotherapy, antibiotics, diets, and physical activity, which may lead to some confounding effects. Second, we are unable to obtain symptoms and signs such as pain, cramping, heaviness, pruritus, and varicose veins at the site of DVT, which can hinder a more comprehensive analysis. Although vascular ultrasonography has gradually replaced venous angiography and is widely used, this method is not the “golden standard”. As a result, the possibility of a false positive or false negative in DVT diagnoses cannot be ruled out in this study. In addition, it was difficult for us to include all variables that may affect the statistical results. The relationship between variables and DVT is correlation, not causation. Therefore, the findings should be interpreted in the context of the clinical situation. Furthermore, no external datasets were used for validation in this study and all models were validated only with cross-validation and a test set during training. Finally, this study lacks comparability with previous studies due to the high variability and lack of sharing of medical data across hospitals. Therefore, the creation of large-scale databases that can be freely accessed plays a vital role in future research and attention should be paid to standardised data management.

## Conclusions

Age, hypertension, fibrinogen, surgical grade, platelets, T triacylglycerol, free fatty acid, d-dimer, globulin ratio, and diabetes are potentially important predictors of DVT. On our data set, the XGBoost model is the most effective in predicting the occurrence of DVT in patients with lower extremity fractures during hospitalization while it still needs external verification research before clinical application.

### Supplementary Information

Supplementary Information.

## Data Availability

The source code is available at 10.5072/zenodo.31925, and the source data is available at 10.5072/zenodo.31921.
