# Supplementary material for: Comparison of different machine learning classification models for predicting deep vein thrombosis in lower extremity fractures
Source: Sci Rep. 2024 Mar 22;14:6901. doi: 10.1038/s41598-024-57711-w (PMC10960026; doi:10.1038/s41598-024-57711-w)
Supplement: Supplementary file 1 — Supplementary Information. [file 41598_2024_57711_MOESM1_ESM.pdf]

Baseline characteristics of lower extremity fractures patients with and without DVT.

Predictors	No-thrombosis group (n = 4217)	Thrombosis group (n = 207)	*P*
Sex [n (%)]			< 0.001
Male	2269 (53.806)	84 (40.580)	
Female	1948 (46.194)	123 (59.420)	
Rh blood type [n (%)]			0.940
Negative	22 (0.522)	1 (0.483)	
Positive	4195 (99.478)	206 (99.517)	
ABO blood type [n (%)]			0.007
O Type Blood	1360 (32.250)	51 (24.638)	
A Type Blood	1647 (39.056)	91 (43.961)	
B Type Blood	929 (22.030)	41 (19.807)	
AB Type Blood	281 (6.664)	24 (11.594)	
Smoking [n (%)]			0.610
No	4089 (96.965)	202 (97.585)	
Yes	128 (3.035)	5 (2.415)	
Alcohol consumption [n (%)]			0.501
No	4103 (97.297)	203 (98.068)	
Yes	114 (2.703)	4 (1.932)	
Operation time [n (%)]			0.611
⩽3h	3848 (91.250)	191 (92.271)	
＞3h	369 (8.750)	16 (7.729)	
Hypoalbuminemia [n (%)]			< 0.001
No	4057 (96.206)	180 (86.957)	
Yes	160 (3.794)	27 (13.043)	
Kidney disease [n (%)]			0.672
No	4133 (98.008)	202 (97.585)	
Yes	84 (1.992)	5 (2.415)	
Cerebrovascular disease [n (%)]			0.168
No	3738 (88.641)	177 (85.507)	
Yes	479 (11.359)	30 (14.493)	
Atrial fibrillation [n (%)]			< 0.001
No	4046 (95.945)	188 (90.821)	
Yes	171 (4.055)	19 (9.179)	
Heart disease [n (%)]			0.658
No	4021 (95.352)	196 (94.686)	
Yes	196 (4.648)	11 (5.314)	
Cancer [n (%)]			0.097
No	3952 (93.716)	188 (90.821)	
Yes	265 (6.284)	19 (9.179)	
COPD [n (%)]			0.112
No	4079 (96.728)	196 (94.686)	
Yes	138 (3.272)	11 (5.314)	
Osteoporosis [n (%)]			0.072

No	3891 (92.269)	198 (95.652)	
Yes	326 (7.731)	9 (4.348)	
Hypertension [n (%)]			0.703
No	3066 (72.706)	148 (71.498)	
Yes	1151 (27.294)	59 (28.502)	
Diabetes [n (%)]			0.073
No	3671 (87.052)	189 (91.304)	
Yes	546 (12.948)	18 (8.696)	
Lung infection [n (%)]			0.288
No	3949 (93.645)	190 (91.787)	
Yes	268 (6.355)	17 (8.213)	
Fracture type [n (%)]			< 0.001
Hip	177 (4.197)	13 (6.280)	
Femur	2551 (60.493)	132 (63.768)	
Tibial	1283 (30.424)	25 (12.077)	
Multiple	206 (4.885)	37 (17.874)	
Surgical grade [n (%)]			nan
I	24 (0.569)	0 (0.000)	
II	155 (3.676)	4 (1.932)	
III	3191 (75.670)	170 (82.126)	
IV	847 (20.085)	33 (15.942)	
Age median [IQR]	60.000 [45.000,76.000]	70.000 [59.000,79.000]	< 0.001
Total cholesterol median [IQR]	3.742 [3.156,4.340]	3.853 [3.230,4.663]	0.012
T triacylglycerol median [IQR]	1.085 [0.856,1.464]	1.204 [0.927,1.550]	0.002
Free fatty acid median [IQR]	0.505 [0.350,0.660]	0.510 [0.340,0.670]	0.723
Albumin median [IQR]	37.060 [33.700,40.230]	34.670 [30.900,37.710]	< 0.001
Globulin ratio median [IQR]	1.420 [1.240,1.620]	1.300 [1.120,1.485]	< 0.001
Calcium median [IQR]	2.240 [2.150,2.320]	2.210 [2.080,2.300]	< 0.001
Potassium median [IQR]	3.880 [3.620,4.140]	3.850 [3.630,4.140]	0.998
Platelets median [IQR]	185.000 [143.000,234.000]	202.000 [153.000,269.000]	0.003
Red blood cells median [IQR]	3.840 [3.280,4.340]	3.470 [2.890,3.990]	< 0.001
White blood cells median [IQR]	8.090 [6.390,10.180]	7.940 [6.220,9.370]	0.067
Fibrinogen median [IQR]	3.320 [2.600,4.240]	3.720 [3.040,4.680]	< 0.001
D dimer median [IQR]	5.860 [2.700,13.100]	6.980 [3.800,14.080]	0.027
INR median [IQR]	1.020 [0.970,1.090]	1.040 [0.970,1.110]	0.036
Prothrombin time median [IQR]	11.800 [11.200,12.500]	12.000 [11.300,12.700]	0.051
Hospital stay median [IQR]	12.000 [8.333,17.000]	12.000 [7.000,17.000]	0.252
C-reactive protein median [IQR]	41.900 [15.830,85.360]	53.500 [20.600,97.410]	0.007
